# Prevalence, awareness, treatment and control of diabetes mellitus among middle-aged and elderly people in a rural Chinese population: A cross-sectional study

**DOI:** 10.1371/journal.pone.0198343

**Published:** 2018-06-01

**Authors:** Qian Wang, Xu Zhang, Li Fang, Qingbo Guan, Liying Guan, Qiu Li

**Affiliations:** 1 Department of Endocrinology, Shandong Provincial Hospital affiliated to Shandong University, Jinan, Shandong, China; 2 Shandong Clinical Medical Center of Endocrinology and Metabolism, Jinan, Shandong, China; 3 Institute of Endocrinology and Metabolism, Shandong Academy of Clinical Medicine, Jinan, Shandong, China; 4 Health Management Center, Shandong Provincial Hospital Affliated to Shandong University, Jinan, Shandong, China; Shanghai Diabetes Institute, CHINA

## Abstract

Diabetes mellitus ranks high on the international health agenda as a global pandemic and as a threat to human health and global economies. A total of 10851 participants aged over 40 years were included in the cross-sectional analysis. This observational study analyzed the prevalence of diabetes mellitus and the awareness, treatment and glycemic control of diabetes in a rural Chinese population. Approximately 25% of middle-aged and elderly rural Chinese residents had diabetes in 2010–2011. The prevalence was higher with older age, dyslipidemia, higher body mass index and larger waist circumference. Among the subjects with diabetes, 40.3% were aware of their condition; 62.9% were receiving treatment, and 16.9% had controlled diabetes. Metformin was the majority oral antidiabetic drug treatment most often prescribed, for either monotherapy or combined therapy. These results indicate that diabetes has become an urgent public health problem in the Chinese rural population because of its high prevalence and low rates of awareness, treatment and control. The management and prevention of diabetes mellitus should be considered an essential strategy at the level of public health.

## Introduction

Diabetes mellitus (DM) ranks highly on the international health agenda as a global pandemic and as a threat to human health and global economies [1]. The International Diabetes Federation (IDF), Diabetes Atlas, shows that there are 425 million people with DM, which reflects a prevalence rate of 8.6% in adults [2]. The conditions of DM vary from country to country worldwide. Since the reform and opening of China, with socio-economic development, lifestyle changes and the aging of its population, DM has become an urgent health issue for the country. Recent studies have shown that the prevalence of DM in China has reached more than 10% among Chinese adults, which is far higher than world average [3]. Worldwide, almost half of people with DM are undiagnosed, but the undiagnosed rate in China is 69.83% [1, 3]. All data indicate a rapid growth rate in DM in China, as a result, an increasing substantial burden in terms of public health and medical care will become apparent in next decades. Therefore, it is urgent to assess the epidemiological characteristics and risk factors of type 2 diabetes and to make elective interventions in a specific population.

There are many complications of DM, such as diabetic retinopathy and diabetic nephropathy, that can lead to blindness and kidney failure[4, 5]. In addition, DM increases the risk of cardiovascular diseases and associated death [6, 7], which causes high expenditures. IDF reported that over 10% of health costs in China could be related to DM [8]. Studies have indicated that the grim situation regarding DM could be mitigated through appropriate management and education [9]. Adequate glycemic control can significantly decrease the risk of DM-related complications, causing a delay in disease progression [10]. National public health management for DM needs to begin right away to delay the progression of DM. However, basic information about the prevalence, treatment and diabetic educational condition should be analyzed to provide evidence for further prevention.

Although a few previous studies have focused on prevalence, awareness, treatment, control and risk-factor assessment in China, the results were inconsistent due to the discrepancy in geographic regions, economy, culture, occupation, diet and life style. Our study aimed to estimate the prevalence, awareness, treatment, and control of DM in a rural area and to show the condition of oral anti-diabetic drug (OAD) therapy.

## Methods

### Subjects and design

From April 2011 to May 2012, data were gathered from the community-based REACTION study, which was created to investigate the epidemiology of metabolic diseases in China [11]. We recruited participants from 11 towns in Ningyang, Shandong. Participants over 40 years old were sought who had lived in the local area for more than 5 years. All the eligible permanent residents from each village in these towns were invited to participate in the study. In total, we enrolled 11,000 subjects in the present study, and 10851 subjects, aged 40 or above, completed the entire study. The study was conducted in the local township public health centers in each town. All the subjects provided consent form before data collection. The study protocol was authorized by the ethics committee of Shanghai Jiao Tong University [11]. The authors had access to information that could identify individual participants during or after data collection. This is a cross-sectional study. We also excluded individuals with missing vital data, such as age and sex (n = 149).

### Data collection

Experienced interviewers administered a well-established questionnaire through a face-to-face interview to collect data on past medical history and lifestyle factors.

Weight, height, and waist circumference (WC) were measured by standardized equipment and procedures and were measured in kilograms or centimeters. WC was measured at the umbilicus level with the participants in the standing position. Weight was measured in light clothes and without shoes. Body mass index (BMI) was equal to weight (kg) divided by squared height (m^2^). Blood pressure was measured in the non-dominant arm three times in succession with a 3-min interval between the measurements with a sitting position. The three readings were further averaged for data analysis.

Fasting blood samples were collected from all participants after fasting for at least 10 hours overnight. Each participant lacking a history of DM was given an oral glucose tolerance test. Hemoglobin A1c (HbA1c) was measured by the Hemoglobin Capillary Collection System (Bio-Rad Laboratories), and the capillary blood samples were shipped and stored at 2°C to 8°C before being measured. The blood samples were shipped in dry ice to the central laboratory of the Shanghai Institute of Endocrine and Metabolic Diseases. Low-density lipoprotein cholesterol (LDL-C), high-density lipoprotein cholesterol (HDL-C), total cholesterol (TC) and triglyceride (TG) were detected by the central laboratory.

### Disease definition

DM was defined as (1) a self-reported previous diagnosis by health care professionals, (2) fasting plasma glucose(FPG) level of 7.0 mmol/L or higher, (3) 2-hour plasma glucose level of 11.1mmol/L or higher. Prediabetes was defined as 6.1 mmol/L≤FPG < 7.0 mmol/L and/or 7.8 mmol/L ≤ 2-hour plasma glucose level < 11.1 mmol/L. Awareness was defined as the proportion of individuals with self-reported physician-diagnosed DM among all participants with DM. Treatment was defined as the percentage of diabetic patients who had taken diabetic medication. Control was characterized as the rate of participants with an HbA1c level under 7.0% among diabetic patients who were treated with diabetic medications.

We defined overweight as a BMI of 24.0 to 27.9, and obesity as a BMI of 28.0 or higher in the participants [12]. When WC was equal to or more than 90 cm in males and 80 cm in females, the participants were defined as having central obesity[12].

### Statistical analysis

Characteristics of the study participants were depicted as the mean (95% CIs) for continuous variables and percentages (95% CIs) for categorical variables among all participants and in subgroups of sex, age, BMI and WC. The χ2 test (for categorical variables) was used to analyze the differences in the control rates with different drugs. A multivariate logistic regression model was used to evaluate risk factors. Adjusted odds ratios were calculated with 95% CIs. Statistical significance was defined as a P value < 0.05. All the statistical analyses were conducted with SPSS version 22.0 software.

## Results

A total of 11000 subjects were investigated in the present study. Of these, 10851 completed the entire study. The general characteristics are shown in Table 1. The mean age of the study subjects was 53.83 years. There were 4441 men (41.1%) and 6410 women (58.9%) (Table 1). DM prevalence increased with age, BMI and WC. The estimated prevalence of prediabetes was 28.9% (95% CI, 28.1%-29.8%) in our participants. (Table 2) The prevalence of DM was estimated to be 24.9% (95% CI, 24.5%-25.4%) in our population, 28.2% (95% CI, 27.5%-28.8%) in men, and 22.7% (95% CI, 22.2%-23.2%) in women (Table 3).

**Table 1:** General characteristics of the rural population in Shandong Province.

	total mean(95% CI)	male mean(95% CI)	female mean(95% CI)
**n**	10851	4440	6411
**BMI(kg/m**^**2**^**)**	25.18(25.09–25.27)	25.03(24.92–25.13)	25.30(25.21–25.39)
**WC(cm)**	87.09(86.34–87.31)	89.42(89.12–89.71)	86.55(86.30–86.80)
**systolic pressure(mmHg)**	138.84(138.41–139.23)	141.30(140.69–141.92)	137.02(136.50–137.55)
**TC (mmol/L)**	5.13(5.10–5.14)	5.05(5.02–5.09)	5.18(5.14–5.20)
**LDL(mmol/L)**	3.03(3.02–3.05)	2.99(2.96–3.02)	3.06(3.08–3.04)
**HDL(mmol/L)**	1.43(1.43–1.44)	1.39(1.38–1.40)	1.46(1.45–1.47)
**TG(mmol/L)**	1.50(1.47–1.52)	1.58(1.56–1.66)	1.45(1.42–1.47)
**Fasting plasma glucose(mmol/L)**	6.36(6.30–6.42)	6.48(6.42–6.54)	6.28(6.23–6.33)
**2-hour plasma glucose(mmol/L)**	9.41(9.35–9.47)	9.51(9.34–9.68)	9.34(9.18–9.47)
**HAb1C(%)**	6.19(6.16–6.23)	6.14(6.10–6.18)	6.22(6.19–6.25)

**Table 2:** The rates of prediabetes within the participants.

	Impaired Fasting Glucose % (95% CI)	Impaired Glucose Tolerance % (95% CI)	Impaired Fasting Glucose and/or Impaired Glucose Tolerance % (95% CI)
**total**	19.2(18.5–20.0)	19.3(25.5–27.5)	28.9(28.1–29.8)
**SEX**			
men	22.0(20.8–23.2)	19.0(18.5–20.0)	30.3(28.9–31.6)
women	17.3(16.4–18.2)	19.5(18.5–20.5)	28.0(26.9–29.1)
**AGE**			
40–49	14.6(13.3–15.8)	18.3(19.7–17.0)	26.1(24.6–27.6)
50–59	19.9(18.6–21.1)	19.3(18.1–20.6)	29.5(28.0–30.9)
60–69	22.7(21.3–24.2)	20.0(18.5–21.4)	31.4(29.7–33.0)
70-	21.6(18.4–24.6)	20.5(17.5–23.6)	28.5(25.1–31.9)
**BMI**			
-23.9	16.9(15.8–18.1)	18.8(17.6–20.0)	27.7(26.4–29.1)
24–27.9	19.8(18.6–21.0)	19.1(17.9–20.3)	28.8(27.5–30.1)
28-	22.0(20.3–23.7)	20.6(18.9–22.2)	31.3(29.4–33.2)
**WC**			
women<80 men<90	17.1(15.9–18.4)	20.0(18.7–21.3)	28.7(27.3–30.2)
women≥80 men≥90	20.3(19.4–21.2)	19.1(18.1–19.9)	29.1(28.0–30.1)

**Table 3:** The estimated prevalence, awareness, treatment and control of diabetes in rural Chinese population.

	Diabetes prevalence % (95% CI)	Awareness % (95% CI)	Treatment % (95% CI)	Control % (95% CI)
**total**	24.9(24.5–25.4)	40.3(39.3–41.2)	62.9 (61.5–64.4)	16.9(15.5–18.3)
**SEX**				
men	28.2(27.5–28.8)	37.5(36.1–38.9)	67.6(65.4–69.8)	19.2(17.0–21.5)
women	22.7(22.2–23.2)	42.7(41.4–44.0)	59.4(57.5–61.4)	15.5(13.6–17.3)
**AGE**				
40–49	17.6(14.6–17.1)	37.4(35.4–39.5)	56.7 (53.3–60.1)	16.8(13.4–20.2)
50–59	24.7(24.0–25.4)	40.6(39.0–42.2)	61.4(58.9–63.8)	17.3(14.9–19.7)
60–69	30.6(29.8–31.4)	40.7(39.1–42.3)	68.4(66.0–70.8)	16.3(14.0–18.6)
70-	35.8(34.0–37.7)	44.0(40.8–47.2)	62.3(57.6–67.0)	22.7(17.6–27.9)
**BMI**				
-23.9	20.5(19.8–21.1)	40.7(39.1–42.4)	59.5(56.9–62.2)	19.6(16.9–22.4)
24–27.9	26.9(26.3–27.6)	40.6(39.2–42.0)	64.1(61.9–66.3)	16.5(14.4–18.6)
28-	29.5(28.5–30.5)	39.2(37.3–41.1)	63.8(60.8–66.8)	16.5(13.6–19.4)
**WC**				
Women<80 Men<90	19.7(19.0–20.4)	34.7(32.9–36.4)	57.1(54.0–60.3)	22.2(18.8–25.7)
women≥80 men≥90	27.8(27.3–28.3)	42.1(41.0–43.2)	64.6(63.0–66.3)	16.9(14.3–17.5)

**Fig 1:**
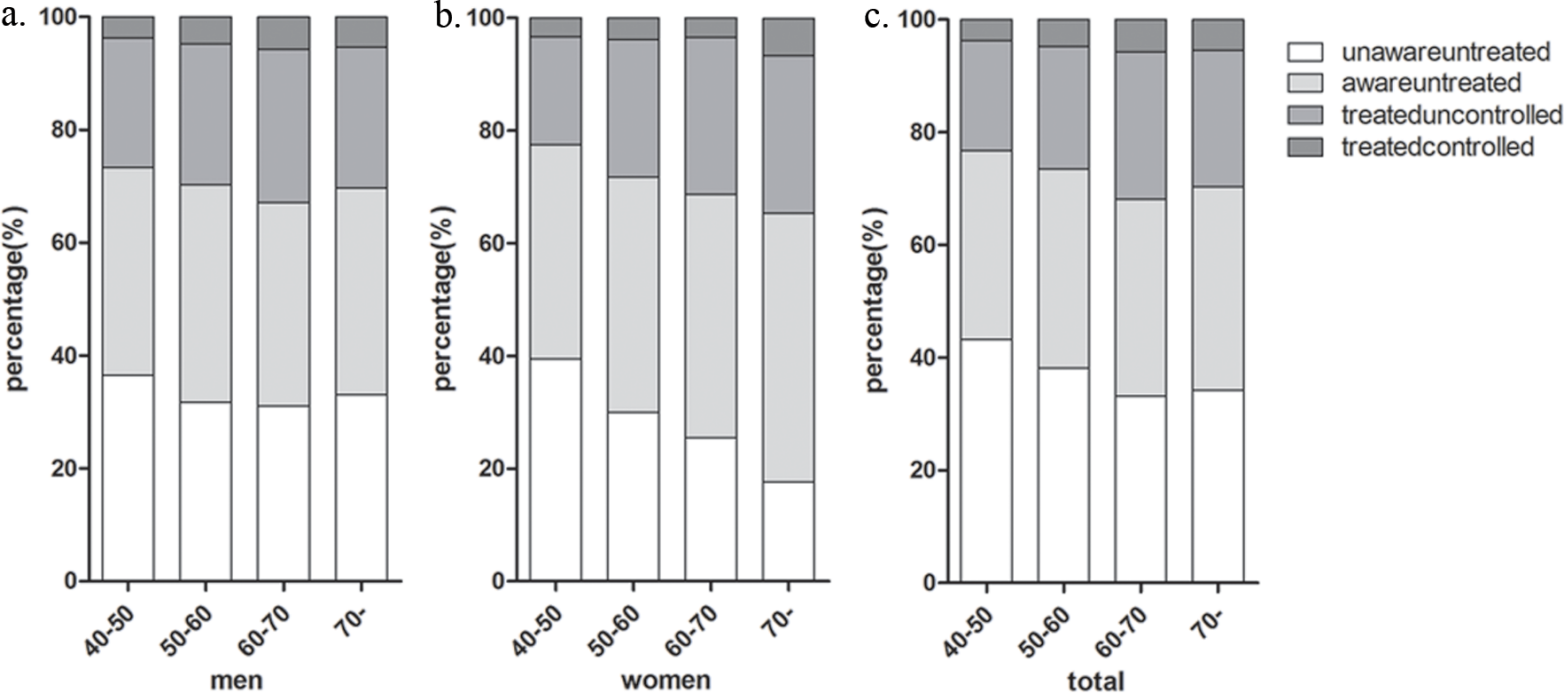
Rates of awareness, treatment, and control of diabetes mellitus by age and sex group. (a). men; (b). women; (c). total.

Among people with DM, 40.3% (95% CI, 39.3%-41.2%) were aware of their condition: 37.5% (95% CI, 36.1%-38.9%) of men and 42.7% (95% CI, 41.4%-44.0%) of women. Among all patients who were aware of their DM, 62.9% (95% CI, 61.5%-64.4%) were treated for this condition: 67.6% (95% CI, 65.4%-69.8%) of men and 59.4% (95% CI, 57.5%-61.4%) in women. Among those patients who were aware of their DM, 16.9% (95% CI, 15.5%-18.3%) had their HbA1c values controlled to a concentration of less than 7.0%: 19.2% (95% CI, 17.0%-21.5%) of men and 15.5% (95% CI, 13.6%-17.3%) of women. The proportion of awareness, treatment and control for DM increased with age to a point, however, it decreased among people in treatment aged > 70 years in treatment. Grouped by age, women with DM showed a decreased trend in the group of patients who were unaware and untreated with increasing age (Fig 1). In other groups of female patients, an increased tendency for a lack of awareness and treatment was shown as the female patients got older. In contrast, male patients showed no variable trend in any of the subgroups divided by awareness, treatment and controlled condition.

Table 4 shows the age-sex-adjusted odds ratios for covariates and DM prevalence, awareness, treatment and control. In the multivariable logistic regression of the risk factors for DM, lower HDL levels, male sex, older age, overweight, obesity, elevated serum total cholesterol, TG, and LDL levels were all significantly associated with a higher risk of DM. No significant relations were shown between those covariates and awareness or treatment. In contrast, LDL, HDL, TC, TG, BMI and WC were significantly associated with control status. Educational levels showed significant association with all the outcomes, including DM prevalence.

**Table 4:** Results of multivariate logistic regression for prevalence, awareness, treatment and control of diabetes.

	diabetes OR (95% CI)	P^1^ value	Awareness OR (95% CI)	P^1^ value	Treatment OR (95% CI)	P^1^ value	Control OR (95% CI)	P^1^ value
HDL	0.57(0.50–0.65)	0.000	0.49(0.39–0.61)	0.000	0.50(0.35–0.73)	0.000	1.02(0.57–1.84)	0.95
LDL	1.24(1.18–1.30)	0.000	0.85(0.78–0.92)	0.000	0.88(0.77–1.00)	0.047	0.64(0.51–0.81)	0.000
TC	1.22(1.17–1.27)	0.000	0.85 (0.79–0.90)	0.000	0.88(0.79–0.97)	0.01	0.78(0.66–0.92)	0.004
TG	1.37(1.32–1.42)	0.000	1.02(0.97–1.06)	0.40	1.03(0.97–1.09)	0.33	0.96(0.85–1.08)	0.48
**BMI**								
-23.9	1		1		1		1	
24–27.9	1.51(1.36–1.67)	0.000	1.00(0.84–1.19)	0.40	1.20(0.90–1.60)	0.21	0.84(0.53–1.32)	0.44
28-	1.81(1.60–2.04)	0.000	0.94(0.76–1.15)	0.98	1.23(0.88–1.72)	0.24	0.83(0.49–1.43)	0.50
WC								
women<80 men<90	1		1		1		1	
Women≥80 men≥90	1.84(1.66–2.04)	0.000	1.31(1.09–1.57)	0.005	1.55(1.14–2.10)	0.005	0.64(0.40–1.01)	0.055
education								
Primary high school and below	1		1		1		1	
High school and above	1.15(1.05–1.26)	0.004	1.25 (1.03–1.52)	0.026	1.59(1.13–2.24)	0.008	1.61(1.07–2.43)	0.024

1.Adjusted for age and/or sex if they were a significant risk factor for prevalence, awareness, treatment and control.

The patterns of OAD treatment are shown in Table 5 among the participants who had been diagnosed with DM. In our research, metformin was the medication most frequently chosen (368 participants, 53.6%), followed by sulfonylureas (140 participants, 20.4%). Acarbose and glinides showed small proportions (1.75%, 1.75%) in the participants receiving OAD treatment. Monotherapy was the treatment for 269 participants (39.2%), most of whom received metformin. No significant discrepancies in control rates were found in different groups, regardless of monotherapy, combined therapy or the number of agents used.

**Table 5:** OAD treatment and control situation in patients treated with diabetes medications.

	number	%	control(n)	control rate(%)	P value
**No. of OADs**					0.261
1	269	39.2	41	15.24	
2	123	17.9	21	17.07	
3	6	0.875	1	16.67	
**OAD**					0.293
metformin	368	53.6	68	18.48	
sulfonylureas	140	20.4	27	19.29	
Acarbose	12	1.75	0	0	
glinides	12	1.75	2	16.67	
monotherapy					0.333
metformin	242	35.3	35	14.29	
sulfonylureas	19	2.77	5	26.32	
Acarbose	3	0.44	1	33.33	
glinides	5	0.73	0	0	

## Discussion

This survey indicates that DM has become a major public health problem in the rural areas of China. Our study estimated that approximately 24.7% of the individuals in our survey may have had DM in 2011. Furthermore, among diabetic patients, it was estimated that 40.3% were aware of their DM and more than half (62.9%) of the people who were aware of their condition were reported to receive treatment for DM. In addition, 16.9% of the patients receiving treatment had adequate control through the treatment. All the data indicate that DM may have become an urgent problem in the rural Chinese population. With the high prevalence of DM, a potential epidemic of diabetes-related complications is just around the corner, including diabetic retinopathy, related cardiovascular disease, and diabetic nephropathy with no effective national intervention to be taken in the future.

The IDF projections predicted that the worldwide prevalence of DM will increase to 8.8% by 2035 [13]. The overall prevalence in the Chinese national survey was 11.6% in the adult population [3], while in our survey, the prevalence of DM in the mid-aged and older aged rural population had climbed to 24.7%. There are possible reasons for the difference: first, our population was somewhat older than those in the national survey. Second, compared with the national survey, the participants in our research were fatter; WC and BMI were both higher in our participants compared with the same age groups in the national survey. The growing prevalence of DM has led to a significant increase in medical costs, not only for the patients but also regarding public health, in the treatment and management of DM and DM-related complications [14, 15]. Common complications can involve many organs and tissues, including both microvascular (neuropathy, nephropathy and retinopathy) and macrovascular disorders (stroke and peripheral vascular disease) [16]. DM increases the risk of cardiovascular diseases and related mortality [6, 7]. Adequate glycemic control can decrease the risk of cardiovascular disease in DM patients [17]. Many studies have indicated that the burden of DM on the public may be reduced through adequate management of DM [9]. Adequate glycemic control can significantly reduce the risk and the progression of DM-related complications [18, 19]. To avoid and ease this social burden, a primary prevention and treatment program addressing DM should be implemented nationally in China.

In 2000–2001, the rates of awareness, treatment and control in the rural areas were 18.77%, 6.18% and 8.02%, respectively, according to a survey with 15236 Chinese adults aged 35–74 years [20], while in our research, the levels of awareness, treatment and control reached 40.3%, 62.9%, 16.9% respectively. The data from another research project in a population from the REACTION study showed values of 36.3%, 27.9%, 34.7%, respectively [21]. Another study focused on the awareness and treatment of diabetes showed respective rates of 43.5% and 36.6% in a population aged 35 or older [22]. The difference between our research and the other studies may result from the discrepancy between the definitions of treatment. In our research, treatment included all the therapies for DM, including dietary therapy while in other studies, prescribed treatment was limited to oral antidiabetic agents and/or insulin injections. The condition of DM is even worse in China than the corresponding condition in a population in India, which showed levels of awareness, treatment and control of 72%, 54%, and 40%, respectively [23]. In the developed countries, for example, the US, the estimates in the elderly population were even higher, with a prevalence of 21% and the rates of awareness, treatment and control being 71%, 51% and 50%, respectively [24]. With a high economic growth rate and the rapid progress of urbanization, many remarkable changes have occurred in social status and lifestyles over the past few years. Chinese consumers have dramatically altered the structure of their diet structure in these years. The percentage of meat and fruit in the diet grew with an increase in supply and quality, while the consumption of grain-based foods decreased [25–27]. In addition, with the coordinated development of industrialization and mechanization, people took part in jobs and transportation requiring less physical activity [25–27]. The change in diet structure and less physical activity lead to the rising rate of metabolic diseases, which might be one of the reasons for the increasing prevalence of DM. Additionally, some potential threat had already appeared in the rural participants. With a rate of prediabetes reaching almost 30%, precautions should be taken immediately to prevent the progression of prediabetes. These results emphasize the overwhelming burden of DM and highlight the need for structured care with defined standards of care for nursing home residents with DM.

The educational level in poor rural areas was often low. Consequently, the individuals lacked health education about DM. As shown in our data, a high educational level was a significant risk for DM, awareness, treatment or control of the condition, which indicated that higher educational level did not correspond to more knowledge and precautions taken concerning DM. The relationships between high educational level and all the conditions may be related to the differences in physical activities between different educational levels which were analyzed in our other research. With this limited knowledge about diabetes, the participants in our study may not adopt effective and suitable measures such as controlling diet or moderate exercise, and this emphasized the need for effective monitoring and management of DM in the rural population, regardless of the level of education. Although the prevention and treatment program in the Chinese diabetes guidelines tends to be standardized, the lack of medical resources in rural areas has led to deficiencies in diabetes education and treatment. People's awareness of the prognosis of diabetes and its complications is far from sufficient, leading to an increasing prevalence of diabetes. In our population, BMI and waist circumference were higher than those in other studies at the same age, suggesting that more education and measures focused on diabetes and its risk factors need to be considered. Since 2016, China has started to implement health services using the community family physician model, which has already been put into practice in the towns in our research. This may greatly promote the education and prevention of diabetes. Rural areas should make full use of such health resources to prevent the further progress of diabetes.

A previous epidemiologic study and our research have shown that serum lipid levels are a risk factor for DM in Chinese populations. In our study dyslipidemia was also a risk factor for diabetic status. Dyslipidemia control in the Chinese DM patients was also rather poor. Previous studies have shown that dyslipidemia is closely related to the condition of glycemic control and that the HbA1c level can be used as the marker of serum lipid level in patients with DM [28]. Our study also suggested that dyslipidemia was a risk for DM. Therefore, for patients with poor glycemic control, in addition to the primary management of DM, attention should be paid to monitor lipid profiles, and appropriate treatment should be given to achieve adequate glycemic control.

Regardless of whether the drug is used, the rate of compliance in our study was kept to a very low level. In addition, whether it was monotherapy or multidrug treatment, the control rates were not significantly different. It was speculated that the low glycemic control rate might be related to the improper selection or poor management and treatment of dyslipidemia. In rural areas, in addition to untreated people and people who were not aware of their DM, people who have started their treatment, should also be widely educated about DM prevention and standardized treatment to improve compliance rate. The current situations regarding of awareness, treatment and control in our study suggest that the integrated management of DM does not have an easy road ahead in China. With all the consideration, elective intervention measures should be taken to reduce the prevalence and to improve awareness, treatment and control of DM in Chinese rural residents. With the increasing prevalence of DM and longer life expectancy in China, the number of patients with DM of long duration in institutions treating DM has increased significantly in recent years. A long term care system for DM should be constructed to delay the progression of the DM and its complications. WHO has also recommended adopting HbA1c as an index to diagnose diabetes in countries and regions where conditions were amenable. However, since HbA1c detection was not yet common in China and the standardization of detection methods was yet sufficient, the instrument and quality control for determining HbA1c cannot meet the requirements of the current diagnostic criteria for diabetes. The use of HbA1c in the diagnosis of diabetes in China is still not recommended in the diabetes guideline[12].

Our research has several strengths. First, it was conducted from a large representative rural population in China. Second, this study used all the three indicators for the diagnosis and evaluation of DM: fasting plasma glucose, 2-hour plasma glucose, and HbA1c, which provides a relatively accurate assessment of the DM situation in rural areas. However, several limitations should also be considered. First, the fact that type 1 and type 2 diabetes were all included in this study may have a certain impact on its results. Second, HbA1c measurement was not determined from venous blood but from a sample from the finger. Venous blood is not suitable; samples for HbA1c measurement must be sent to Shanghai for centralized detection, and venous blood, which lasts for slightly less than a week, does not store well in the EDTA tubes for longer durations. Finally, our research enrolled a middle-aged and elderly population which cannot represent the whole population of the rural region in China.

## Conclusion

Our research indicated that prevalence of DM was high and increasing in the rural Chinese population. Furthermore, aspects of DM status such as awareness, treatment, and control are not looking positive. DM may have reached an urgent situation in the mid-aged and elderly rural population. More attention and measurement should be given to improve the overall management of DM and to prevent DM progression and associated complications in rural areas.

## Supporting information

S1 Table Detailed characteristics of the rural population in Shandong Province. (DOCX)
