# Supplementary material for: Prevalence, awareness, treatment and control of diabetes mellitus among middle-aged and elderly people in a rural Chinese population: A cross-sectional study
Source: PLoS One. 2018 Jun 1;13(6):e0198343. doi: 10.1371/journal.pone.0198343 (PMC5983453; doi:10.1371/journal.pone.0198343)
Supplement: S1 Table — (DOCX) [file pone.0198343.s001.docx]

S1 table detailed characteristics of the rural population in Shandong Province

|  | **n** | **BMI**  **(kg/m^2^)** | **WC(cm)** | **systolic pressure(mmHg)** | **TC (mmol/L)** | **LDL**  **(mmol/L)** | **HDL**  **(mmol/L)** | **TG**  **(mmol/L)** | **Fasting plasma glucose**  **(mmol/L)** | **2-hour plasma glucose**  **(mmol/L)** | **HAb1C**  **(%)** |
| --- | --- | --- | --- | --- | --- | --- | --- | --- | --- | --- | --- |
| **total** | 10851 | 25.18(25.09-25.27) | 87.09(86.34-87.31) | 138.84(138.41-139.23) | 5.13(5.10-5.14) | 3.03(3.02-3.05) | 1.43(1.43-1.44) | 1.50(1.47-1.52) | 6.36(6.30-6.42) | 9.41(9.35-9.47) | 6.19(6.16-6.23) |
| **SEX** |  | | | | | | | | | | |
| men | 4440 | 25.03(24.92-25.13) | 89.42(89.12-89.71) | 141.30(140.69-141.92) | 5.05(5.02-5.09) | 2.99(2.96-3.02) | 1.39(1.38-1.40) | 1.58(1.56-1.66) | 6.48(6.42-6.54) | 9.51(9.34-9.68) | 6.14(6.10-6.18) |
| women | 6411 | 25.30(25.21-25.39) | 86.55(86.30-86.80) | 137.02(136.50-137.55) | 5.18(5.14-5.20) | 3.06(3.08-3.04) | 1.46(1.45-1.47) | 1.45(1.42-1.47) | 6.28(6.23-6.33) | 9.34(9.18-9.47) | 6.22(6.19-6.25) |
| AGE |  | | | | | | | | | | |
| 40-49 | 3195 | 25.65(25.52-25.77) | 87.40(87.05-87.76) | 131.10(130.45-131.74) | 4.91(4.87-4.94) | 2.86(2.83-2.89) | 1.40(1.39-1.41) | 1.56(1.40-1.61) | 6.06(6.00-6.12) | 8.49(8.32-8.67) | 5.95(5.62-6.24) |
| 50-59 | 3950 | 25.25(25.14-25.36) | 87.71(87.40-88.02) | 138.65(138.00-139.30) | 5.18(5.14-5.21) | 3.06(3.04-3.09) | 1.44(1.43-1.45) | 1.48(1.45-1.52) | 6.43(6.37-6.50) | 9.31(9.13-9.49) | 6.23(6.19-6.27) |
| 60-69 | 3031 | 24.81(24.68-24.94) | 87.96(87.59-88.33) | 145.20(144.42-145.98) | 5.29(5.25-5.33) | 3.17(3.14-3.20) | 1.46(1.44-1.47) | 1.47(1.43-1.50) | 6.55(6.48-6.62) | 10.33(10.09-10.56) | 6.35(6.30-6.39) |
| 70- | 675 | 24.33(24.03-24.62) | 88.45(87.60-89.30) | 147.98(146.29-149.67) | 5.15(5.06-5.24) | 3.05(2.99-3.12) | 1.42(1.39-1.45) | 1.48(1.39-1.57) | 6.56(6.42-6.71) | 10.84(10.39-11.30) | 6.37(6.27-6.46) |
| BMI |  | | | | | | | | | | |
| -23.9 | 4182 | 21.70(21.65-21.75) | 81.26(81.01-81.51) | 135.54(134.88-136.20) | 5.01(4.97-5.02) | 2.90(2.87-2.92) | 1.53(1.52-1.55） | 1.18(1.15-1.20) | 6.20(6.13-6.26) | 8.95(8.76-9.11) | 6.08(6.04-6.12) |
| 24-27.9 | 4384 | 25.89(25.86-25.92) | 89.04(88.80-89.28) | 139.94(139.32-140.56) | 5.19(5.15-5.22) | 3.10(3.08-3.13) | 1.39(1.38-1.40) | 1.61(1.57-1.65) | 6.44(6.38-6.49) | 9.67(9.48-9.83) | 6.22(6.19-6.26) |
| 28- | 2192 | 30.34(30.25-30.42) | 96.70(96.31-97.09) | 142.61(136.85-148.61) | 5.21(5.16-5.26) | 3.13(3.09-3.16) | 1.32(1.30-1.34) | 1.90(1.82-1.97) | 6.54(6.46-6.62) | 9.82(9.57-10.05) | 6.33(6.28-6.38) |
| WC |  | | | | | | | | | | |
| women＜80  men＜90 | 3592 | 22.80(22.70-22.89) | 77.94(77.73-78.15) | 136.61(135.89-137.33) | 5.02(4.97-5.05) | 2.93(2.90-2.96) | 1.52(1.50-1.54) | 1.23(1.19-1.26) | 6.13(6.07-6.19) | 8.71(8.53-8.87) | 6.00(5.96-6.04) |
| women≥80  men≥90 | 7055 | 26.41(26.34-26.49) | 92.71(92.52-92.89) | 139.84(139.36-140.33) | 5.17(5.14-5.20) | 3.08(3.06-3.10) | 1.38(1.37-1.39) | 1.64(1.61-1.67) | 6.49(6.44-6.53) | 9.82(9.66-9.95) | 6.28(6.25-6.31) |
